# Activation of CamKIIα expressing neurons on ventrolateral periaqueductal gray improves behavioral hypersensitivity and thalamic discharge in a trigeminal neuralgia rat model

**DOI:** 10.1186/s10194-021-01257-z

**Published:** 2021-05-27

**Authors:** K. C. Elina, Byeong Ho Oh, Jaisan Islam, Soochong Kim, Young Seok Park

**Affiliations:** 1 Department of Neuroscience, College of Medicine, Chungbuk National University, Cheongju, South Korea; 2 Department of Neurosurgery, Chungbuk National University Hospital, Cheongju, South Korea; 3 Department of Veterinary Medicine, Chungbuk National University, Cheongju, South Korea

**Keywords:** Microdialysis, Optogenetics, Periaqueductal gray, Thalamus, Trigeminal neuralgia

## Abstract

**Background:**

Preceding studies have reported the association of chronic neuropathic orofacial pain with altered ongoing function in the ventrolateral periaqueductal gray (vlPAG). However, its role in trigeminal neuralgia (TN) lacks attention. We here reported the aspect that vlPAG neurons play in TN nociceptive processing by employing excitatory neuron-specific optogenetic approaches.

**Methods:**

TN was generated via unilateral infraorbital nerve chronic constriction in Sprague Dawley rats which induced mechanical and thermal pain sensitivity in air puff and acetone test, respectively. Channelrhodopsin conjugated virus with CamKIIα promoter was used to specifically activate the excitatory vlPAG neuronal population by optogenetic stimulation and in vivo microdialysis was done to determine its effect on the excitatory-inhibitory balance. In vivo extracellular recordings from ventral posteromedial (VPM) thalamus were assessed in response to vlPAG optogenetic stimulation. Depending on the experimental terms, unpaired student’s t test and two-way analysis of variance (ANOVA) were used for statistical analysis.

**Results:**

We observed that optogenetic activation of vlPAG subgroup neurons markedly improved pain hypersensitivity in reflexive behavior tests which was also evident on microdialysis analysis with increase glutamate concentration during stimulation period. Decreased mean firing and burst rates were evident in VPM thalamic electrophysiological recordings during the stimulation period. Overall, our results suggest the optogenetic activation of vlPAG excitatory neurons in a TN rat model has pain ameliorating effect.

**Conclusions:**

This article presents the prospect of pain modulation in trigeminal pain pathway via optogenetic activation of vlPAG excitatory neurons in rat model. This outlook could potentially assist vlPAG insight and its optogenetic approach in trigeminal neuropathic pain which aid clinicians endeavoring towards enhanced pain relief therapy in trigeminal neuralgia patients.

## Introduction

Irrespective of the numerous therapeutic approaches, trigeminal neuralgia (TN) is still deemed a valid question to treatment. TN is one of the most debilitating chronic neuropathic disorders resulting from injury or compression of the trigeminal nerve or its branches [1]. It is depicted by emotionally unpleasant intense pain episodes originating from nerve branches [2]. TN patients suffer from light contact and thermal hypersensitivity that prompts a pain attack, impedes the patient’s ability to perform routine tasks, and creates a regression in life quality [3]. The infraorbital nerve constriction is a validated experimental rat model for the development of allodynia in the ipsilateral vibrissae area [4] as this model has numerous characteristics that resemble clinical disorders in humans struggling with TN [5].

The periaqueductal gray (PAG) is a crucial midbrain structure involved in a wide variety of functions [6, 7] and serves as a gateway within the endogenous analgesic network, which suppresses nociceptive ascending signals. PAG is divided into rostro-caudally running longitudinal columns and two of these columns serve distinctive functions in modulating pain responses. The ventrolateral column of the PAG (vlPAG) is of significance to trigeminal nociceptive modulation. The contribution of vlPAG glutamatergic neuron in itching and pain activity has been reported [8]; in addition there are experimental supports concerning its bidirectional role of excitatory and inhibitory neurons in nociception [9]. However, these studies are mainly focused on spinal pain modulation and less interest has been given to orofacial pain modulation. Previous animal research reports have addressed the electrical stimulation of the PAG inhibiting the trigeminal nociceptive input; thus, PAG dysfunction might lead to the disinhibition of trigeminal afferents [10]. The reduced gray matter volume in the PAG further confirmed that anatomic impairments in this region might underpin TN pathogenesis [11]. For patients with severe, intractable facial pain that is refractory to other treatments, deep brain stimulation (DBS) of PAG has been considered a potential therapeutic option [12] but lack of specificity could activate other neuronal populations that trigger effects other than analgesia. It is tempting to speculate that output from the vlPAG has a purely analgesic action however it comprises diverse subpopulations of neurons with distinct neurochemical properties that regulate excitatory and inhibitory neurotransmission. With the use of optogenetics, better temporal specificity can be achieved to target glutamatergic populations in the vlPAG [13–15]. With the constriction of the trigeminal nerve root, somas located in the trigeminal ganglion send signals to the PAG and project to thalamic nuclei. Pain signals from the PAG project to the sensory thalamus and project to higher cortices. The thalamus also has reciprocal interactions with motor cortex parts and the PAG. Applying optogenetic stimulation at the vlPAG might act on the pain inhibitory pathway and most likely results in a pain relief effect. Principally, trigeminal activation has been documented to alter the PAG’s functional activity as an important midbrain control site for descending pain inhibitory systems. The vlPAG has a vital regulating role in nociceptive transmission with the projection neurons in trigeminal terminals [16]. However, the optogenetic modulation of the vlPAG in TN remains to be fully elucidated.

Currently, there are no studies investigating optogenetic effects in vlPAG glutamatergic neurons in relation to TN. The primary objective of our study is to explore the alterations in sensory pain behaviors in a TN rat model under optogenetic stimulation in vlPAG excitatory neurons and its influence on neuronal firing activity in the sensory thalamus. Furthermore, we hypothesized the modulation of extracellular gamma-aminobutyric acid (GABA) and glutamate level in vlPAG occurs in response to optic stimulation in vlPAG.

## Materials and methods

### Animals and housing

The study comprised sixty female Sprague Dawley rats (8 weeks age; 200–250 g on arrival; Koatech, Pyeongtaek, South Korea) housed in a temperature- and humidity-controlled conventional area (20 °C; 30% humidity) with a 12/12-h light-dark period. Fresh chow and water were supplied ad libitum. All animal experiments were performed in a randomized, double-blind, monitored manner during the light hours. All animal experiments were performed within the Laboratory Animal Research Center, Chungbuk National University***.***

**Fig. 1:**
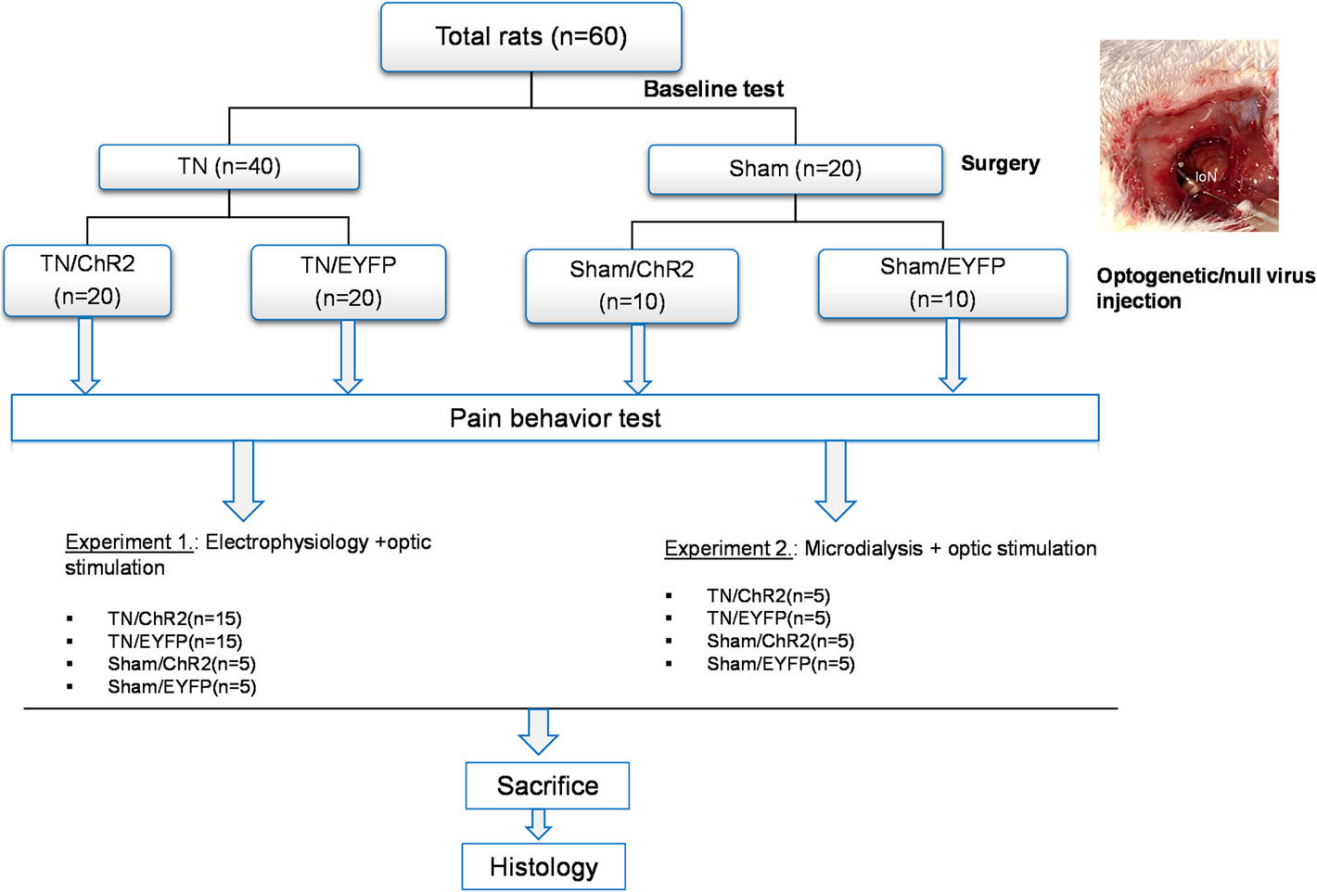
Experimental flowchart

The experimental flowchart is presented in Fig. 1.

### Neuropathic pain model

Rats were randomly allocated to unilateral infraorbital nerve (IoN) constriction (*n* = 40) or sham surgery (*n* = 20). We followed the procedure as described in other articles [17, 18]. The combination of 15 mg/kg zoletil (Zoletil50®, Virbac Laboratories, Carros, France) and 9 mg/kg xylazine (Rompun®, Bayer AG, Leverkusen, Germany) in saline was employed intraperitoneally for anesthesia purposes. Then, animals were placed in a sterile surgical field, and hair near the infraorbital area was shaved and scrubbed with alcohol and betadine. Eye ointment was applied to both eyes to prevent drying, and the animal’s head was placed in a stereotaxic frame. A curved scalp incision 2 mm above the eye region was made by exposing the skull and nasal bone. A cotton swab was used to soak up blood, and with the help of Dumont forceps, blunt dissection was performed separating the IoN from the surrounding connective tissue and muscle. The 1 ml syringe needle was curved, blunted, and slipped under the IoN for easy grasping and ligation. Two loose ligations were made with a 3–0 silk suture around the right IoN with a gap of 2 mm. Then, the incision was sealed with silk (3–0). Sham animals underwent the same procedure except that they did not undergo ligature.

### Stereotaxic viral injections

Following IoN constriction, animals were placed on a stereotaxic apparatus, and a viral vector was infused into the respective group following previous protocol [19]. TN and sham rats were randomly further allocated into two subgroups. Twenty rats in the TN groups were subjected to optogenetic viral vector injection, while the remaining rats were subjected to null virus injection. Similarly, ten sham-operated animals received optogenetic viral vectors, and the other ten received a null virus. We used adeno-associated virus (AAV) serotype 2 with human channelrhodopsin (hChR2) mutated at H134R and fused to enhanced yellow fluorescent protein (EYFP) driven by CaMKIIα promoter for optogenetic activation [AAV2-CamKIIα-hChR2(H134R)-EYFP] (Korea Institute of Science and Technology, Seoul, Republic of Korea). The concentration of the optogenetic viral vector was 1.9 × 10^13^ GC/μL. We used AAV2-CamKIIa-EYFP as a null virus with a concentration of 5 × 10^12^ GC/μL. The respective virus was unilaterally injected into the contralateral vlPAG (stereotaxic coordinates from bregma: anterioposterior, AP = -8 mm; mediolateral, ML = − 0.6 mm; dorsoventral, DV = − 5.6 mm) at a rate of 0.4 μl/min for 5 min with a Hamilton syringe and an automated microsyringe pump (KD Scientific Legato® 130 Syringe Pump, Harvard Apparatus, Holliston, MA, USA). The needle of the syringe was held in the same position for 5 min to prevent backflow and was slowly retracted. The incised area was sutured to close the wound. The animals were maintained at sternal recumbency and kept in separate cages. The stereotaxic atlas [20] was used to determine the coordinates for cannulation and injections.

### Behavioral assessment for pain

The reflexive behaviors in response to mechanical and thermal stimulation were measured a day before (baseline test) the IoN constriction or sham operation and on days 3, 7, 15 and 21 post-operation.

Air puff test is used to quantify the mechanical allodynia of the orofacial region in the animal model of TN [21]. It is based on face withdrawal behavior in response to constant air puffs of graded pressures that are applied to the affected orofacial area. For behavioral observation, each animal in the three groups was placed in rodent Panlab holders (Scitech Korea Inc., Seoul, South Korea). The animals were habituated for at least 15 min, and the test was performed in a darkened and noise-free room. An escape from the air puff or aggressive actions, such as biting, were considered withdrawal behavioral responses after the application of constant air puff pressure for a duration of 4 s to the vibrissal pad territory of each side with a 10 s interval between puffs. The air puff pressure and intervals were regulated by a pneumatic pump module (BH2 system, Harvard Apparatus, USA). Air puffs were introduced through a metal tube of 26 gauges (length, 10 cm) placed at a 90 cm angle from the skin. The air puff cutoff was 40 psi (pounds per square inch) [22]. Compared to baseline values, a substantial decrease in the air puff threshold was described as mechanical allodynia.

For the behavioral test of cold allodynia, animals were placed in Plexiglass cages and habituated for 10 min. Thereafter, few drops of 99.7% acetone were placed onto the vibrissal pad area, and the number of episodes of scratching and aggressive actions involving extreme head shaking was noted for 2 min. Rubbing actions involving body parts other than the face were excluded from evaluation [23]. At intervals of 5 min, we repeated the behavioral test three times, and the number of responses was averaged. Cold allodynia was inspected at 3, 7, 15, and 21 days after surgery.

### Cannula implantation

The cannula implantations were done under 15 mg/kg tiletamine/zolazepam and 9 mg/kg xylazine anesthesia.

For optic cannula implantation, we fixed three anchor screws to the skull and then stereotaxically mounted an optic fiber (MFC_200/230–0.48_###_ZF2.5_A45, Doric Lenses, Quebec City, Quebec, Canada) cut into a length of 5.3 mm to adjust the vlPAG length. Optic cannula was implanted [24, 25] into the vlPAG contralateral to the IoN (AP = -8 mm, ML = − 0.6 mm, DV = − 5.3 mm) followed by firm fixation with super bond and dental cement (Ortho-jet Pound Package, Lang Dental, Wheeling, IL, USA) simultaneously. The animals were kept in recovery cages to rest following implantation.

Another group of neuropathic(*n* = 10) and sham(*n* = 10) rat models were subjected to guide cannula implantation for microdialysis 3 days before the experiments. A longitudinal incision was made along the midline of the skull to expose bregma and lambda. The guide cannula (CXGF-5; Eicom) was implanted above the vlPAG (from bregma: AP − 8 mm, ML − 0.6 mm, DV − 5 mm at an angle of 10°) and sealed with dummy cannula (CXDF-5) until the experiment to avoid fluid loss.

### Behavioral test under laser stimulation

One-week post cannula implantation, vlPAG neurons in the right hemisphere of the brain were exposed to a blue laser (473 nm, 10 mW output at the tip of the 200 μm fiber, frequency = 20 Hz, pulse width = 4 ms) in all the groups. Blue light was delivered from the 473-nm laser (BL473T3–100, ADR-700D, Shanghai, China) via the optical fiber through a rotary joint patch cable that enables movement during testing. The optical commutator was connected to an optic fiber cable that was connected to a surgically implanted zirconia ferrule for in vivo optical stimulation [26] of the target area. The laser parameters were controlled by a waveform generator (Keysight 33511B-CFG001, Keysight, Santa Rosa, CA, USA). Rats were subjected to mechanical and thermal behavioral tests under optogenetic stimulation (laser on vs laser off). All behavioral tests were performed after 30 min of adaptation to the test room.

### In vivo extracellular recording

Three weeks after virus injection, both TN and sham rats were subjected to in vivo extracellular recording. Extracellular recordings were acquired from the right ventromedial thalamic nucleus coordinates: AP, − 3.5 mm; ML, − 2.8 mm; DV, 6.0 mm from bregma using a quartz-insulated carbon electrode (E1011–20, Carbostar-1, Kation Scientific, Minneapolis, MN, USA). The recordings were performed under general anesthesia (a mixture of Zoletil 50 and Rompun) inside a Faraday cage with dim lighting. Spontaneous and evoked neural activity in the VPM was monitored. The electronic interface board (EIB-36, Neuralynx, USA) was connected to 36 channel headstage and preamplifiers, and the outputs were transferred to a Cheetah Acquisition System (Neuralynx, USA). Neuronal signals were filtered, amplified, and sampled at 30,303 Hz. We digitized (40 kHz) and band filtered (0.9–6 kHz) the waveforms via a Digital Lynx SX data-acquisition system (Neuralynx, Bozeman, USA). The units were sorted off-line with Neuralynx’s Spikesort 3D software. Waveforms with separable clusters and consistent shapes were identified as single units and exported to NeuroExplorer® (Neuralynx Inc., Montana, USA) for data analysis. Then, these waveforms were analyzed by generating rate histograms at a rate of bursts per second. Under different light conditions, the rate histograms (spikes/seconds) of lesioned animals were explored. Burst analysis was performed following an established protocol using NeuroExplorer [9].

### In vivo microdialysis

Microdialysis is a minimally invasive sampling technique that enables the rapid, in vivo collection of neurotransmitters such as glutamate and GABA at different time points following injury [27, 28]. To combine optical stimulation with simultaneous microdialysis in the vlPAG, an optogenetics-compatible microdialysis probe (CX-F-05–01; Eicom, Japan) was used. On the day of analysis, the dummy cannula was replaced with a dialysis probe. The inlet and outlet of the probe were connected to a two-channel fluid swivel system (SSU-20, Eicom, Japan), and artificial cerebrospinal fluid (ACSF, Tocris Bioscience, Bristol, UK) was infused through the probe using an infusion pump (ESP-32, Eicom, Japan) at a rate of 0.5 μL/min. Under anesthetized and unrestrained settings, microdialysis was performed in testing cage. After an equilibrium for 2 h period, 10 μL dialysate was collected every 20 min into vials placed on fraction collector whose temperature was regulated by electronic cooler (EFR-82, Eicom, Japan). The samples were collected under three optic conditions- pre, stim and post. During stimulation period at fraction 4 and 5(30–50 min), blue laser was turned on whereas turned off at pre- and post-optical conditions. The samples were immediately removed following collection and stored at − 80 °C until assay. After completion of microdialysis, the animals were decapitated and macroscopically tested the proper localization of the probes in the vlPAG. In the data analysis, only rats correctly implanted with the probes were included.

### GABA and glutamate detection

Liquid chromatography–mass spectrometry (LC–MS) was used to detect GABA and glutamate concentration in vlPAG during laser on and off conditions in TN and sham rats. As internal standards, Glu-d5 (PHR1107-1G, L-Glutamic acid, Sigma-Aldrich Co.,St. Louis, MO, USA) and GABAd6 (03835, γ-Aminobutyric acid, Sigma-Aldrich Co.,St. Louis, MO, USA) were used.

On an Agilent 1100 high-performance liquid chromatography (HPLC) system (Agilent Technologies, Santa Clara, CA, USA) comprising a G1322A degasser, a G1311A quaternary pump, a G1313A well-plate autosampler, and a G1316A thermostated column compartment, chromatographic analysis was performed. For MS detection, a G1946D mass spectrometer (Agilent Technologies, CA, USA) prepared with an electrospray source interface was utilized. For LC/MS identification, data acquisition and analysis were carried out using Agilent ChemStation (version B.02.01). Chromatographic separation was achieved on a column of Agilent XDB-C18 (3.0 mm × 50 mm; id, 1.8 μm) and eluted with a mobile phase of acetonitrile: 0.1% aqueous solution of formic acid (24:76, v/v) at a flow rate of 0.3 mL/min. The temperature of the column was maintained at 25 °C, the autosampler was maintained at 4 °C and the volume was 5 μL. 4.5 min per sample was the measurement time. The HPLC device was linked through an electrospray ionization (ESI) interface to the mass spectrometer. Selected ion monitoring was used, and the fragmentation transitions were m/z 511.1 for curculigoside and m/z 579.1 for naringin.

### Histology

Animals were anesthetized with a zoletil/xylazine combination, and a transcardial perfusion [29] was performed with phosphate buffer solution (PBS) followed by 4% paraformaldehyde (PFA) solution. Brains were extracted and fixed in 4% PFA overnight followed by immersion in 30% sucrose solution before embedding. In the optimum cutting temperature (OCT, Tissue Tek®, Sakura, USA) compound, we embedded brain tissues followed by cryofreezing with liquid nitrogen and isopentane. The frozen samples were stored at − 80 °C until use. Coronal brain tissue (20 μm) sections were made using a cryostat (Thermo Scientific, Waltham, MA, USA). To locate vlPAG and electrode sites, sectioned slides were also stained with cresyl violet staining. The brain sections were incubated with DAPI (Vectashield®, Vector Laboratories, Inc. Burlingame, CA 94010) and mounted with coverslips. Viral expression was visualized on the slides under a fluorescence microscope**.** The images were acquired using cellSens Standard (Olympus Corp., Tokyo, Japan) software and merged employing ImageJ software (National Institutes of Health, MD, USA).

### Statistics

Data are represented as the mean ± standard deviation (SD) and were compared via either an unpaired t test, two-way analysis of variance (ANOVA) with Tukey’s post hoc test, or a repeated-measures ANOVA reliant on the experiment terms. All data were evaluated using GraphPad Prism (GraphPad Software version 8.4.2, Inc., San Diego, CA, USA). *P* < 0.05 was the significance threshold in all conditions.

## Results

### Excitatory vlPAG neurons express ChR2

**Fig. 2:**
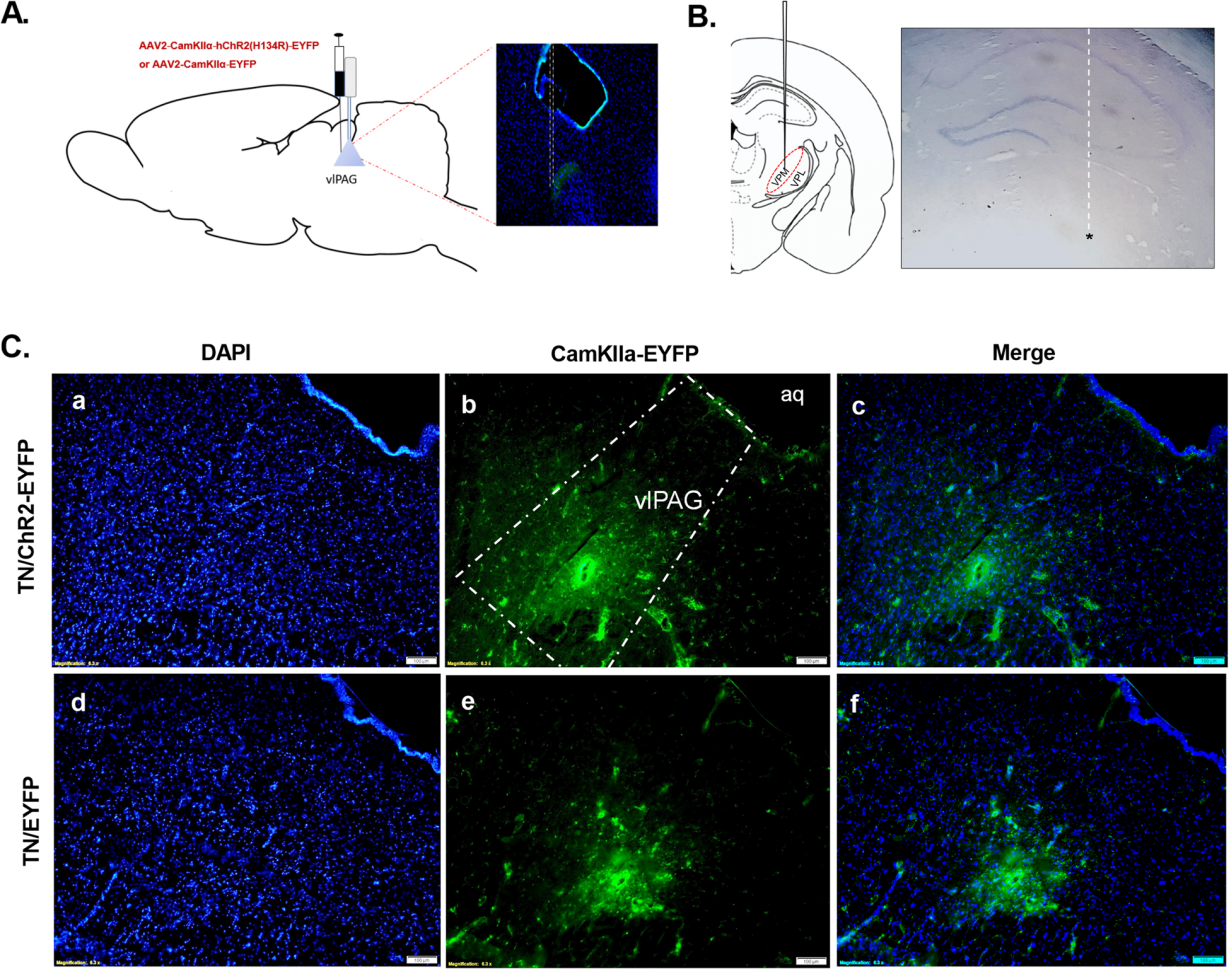
Expression of viral vector in vlPAG. **a** Schematic and immunofluorescent diagram showing the location of virus injection and optic stimulation in vlPAG. **b** Schematic and cresyl violet stained diagram showing the location of electrode in VPM thalamus. **c** Immunofluorescence reveals the optogenetic viral expression in the vlPAG of TN/ChR2(above) and TN/EYFP animals (below); DAPI (**a, d**), EYFP (**b, e**), Merge (**c, f**). Scale bar = 100 μm in all images

We observed the transfection of an AAV vector in our desired target “vlPAG”, which was stereotaxically identified with the Paxinos and Watson rat brain atlas in both optogenetic and null groups. CaMKII promoter conjugated in AAV virus specifically aim at the excitatory neurons in the target area. Figure 2a shows the schematic sagittal section of vlPAG where virus was injected, and light was stimulated. The placement of recording electrodes in the VPM is illustrated by the schematic (left) and cresyl violet-stained (right) coronal portion (Fig. 2b). Immunofluorescent images confirmed the viral expression in vlPAG in TN/ChR2 and TN/EYFP rats (Fig. 2c).

### Variation in pain behaviors following IoN-CCI

**Fig. 3:**
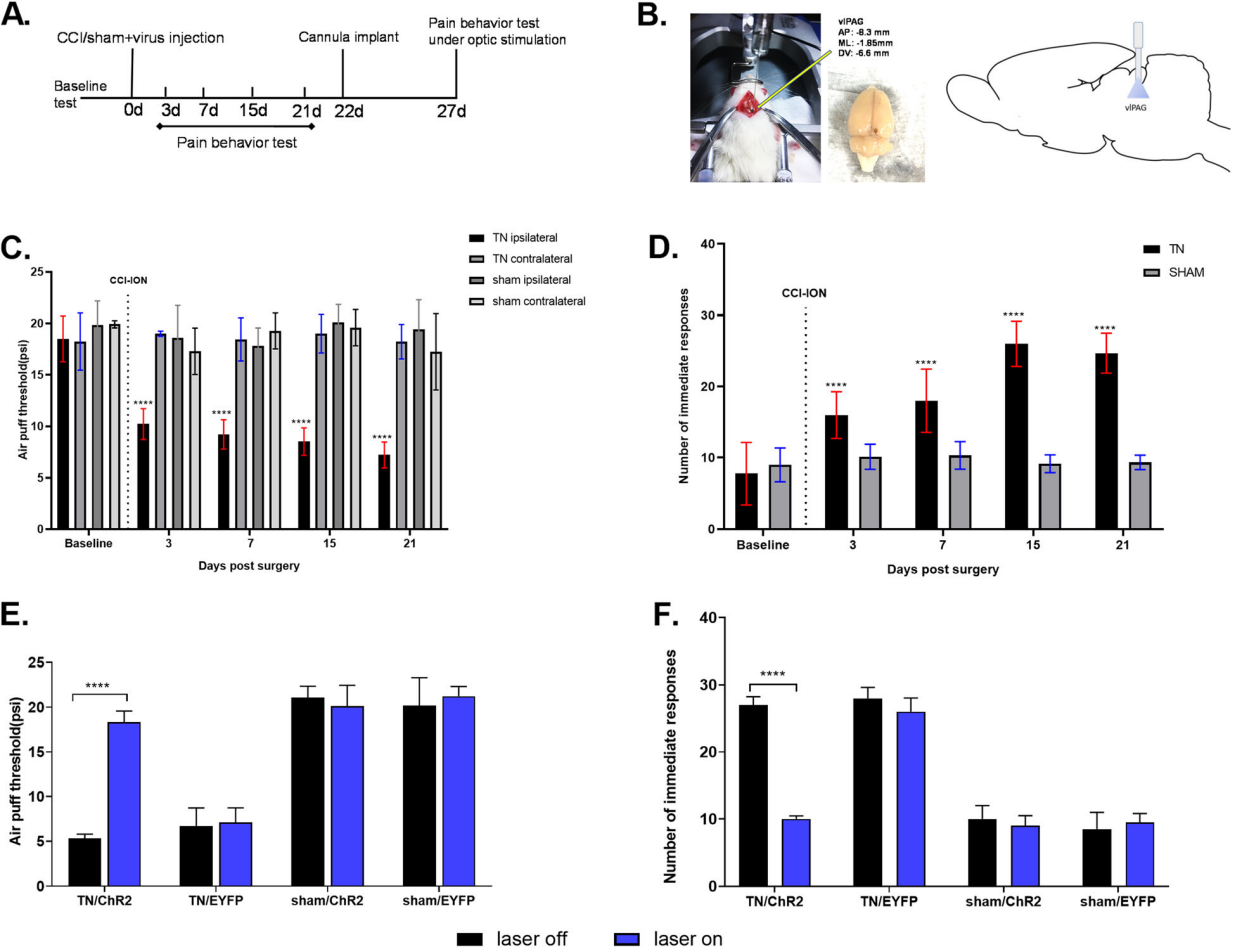
Pain behavioral alteration observed in TN and sham groups. **a** Experimental design for pain behavior test and cannula implantation in animal models (**b**) Diagram showing area coordinates for virus injection and optic stimulation. **c** Air-puff test results for the ipsilateral and contralateral trigeminal facial area. **d** Cold hyperalgesia results for the ipsilateral facial area following treatment with acetone drops. **e** Air-puff test scores for the ipsilateral trigeminal facial region under optic stimulation. **f** Number of immediate responses at the ipsilateral facial area following treatment with acetone drops under optogenetic stimulation in vlPAG. ****, *p* < 0.0001, significant difference determined via two-way analysis of variance (ANOVA)

The infraorbital nerve chronic constriction injury (CCI) has been developed and characterized as an experimental model that reproduces important aspects of TN in humans. The IoN-CCI induces mechanical and thermal hypersensitivity that occurs across different time courses. Following chronic constriction of the IoN and viral injection, behavioral pain differences was observed (Fig. 3a and b) which suggested the induction of chronic pain occur in TN animals in contrast to the pain experienced by sham-operated animals. In the TN group, a significant gradual decline was observed in the air puff test threshold over 21 days on the ipsilateral side (18.50 ± 2.23 psi to 7.20 ± 1.26 psi; two-way ANOVA, *F* (4, 580) =40.42, *p* < 0.0001, Fig. 3c). In addition, the number of responses significantly increased in TN rats in the acetone drop test from 7.76 ± 4.38 to 24.68 ± 2.80 (two-way ANOVA, F (4,290) =71.77, *p* < 0.0001, Fig. 3d). These results are tabulated in Table 1 and are consistent with previous findings [22, 30].

**Table 1:** Mechanical and thermal orofacial pain sensitivity tested by air puff test and acetone drop on trigeminal neuralgia and sham rat models

	Air puff Threshold (psi)				
Animal groups	***Baseline***	***Day 3***	***Day 7***	***Day 15***	***Day 21***
*TN ipsilateral*	18.50 ± 2.23	10.21 ± 1.50****	9.20 ± 1.43****	8.50 ± 1.34****	7.20 ± 1.26****
*TN contralateral*	18.35 ± 2.89	19 ± 0.25	18.45 ± 2.10	19.01 ± 1.88	18.23 ± 1.67
*Sham ipsilateral*	19.36 ± 2.35	18.57 ± 3.19	17.81 ± 1.75	20.12 ± 1.74	19.42 ± 2.90
*Sham contralateral*	19.12 ± 0.35	17.29 ± 2.25	19.29 ± 1.75	19.59 ± 1.77	17.25 ± 3.72
	**Cold hyperalgesia (No. of responses)**				
	***Baseline***	***Day 3***	***Day 7***	***Day 15***	***Day 21***
*TN*	7.76 ± 4.38	15.98 ± 3.28****	18 ± 4.45****	25.98 ± 3.17****	24.68 ± 2.80****
*sham*	9 ± 2.34	10.12 ± 1.75	10.31 ± 1.93	9.14 ± 1.25	9.33 ± 1.01

*TN* Trigeminal neuralgia model (*n* = 40), *sham* sham lesioned (*n* = 20), *psi* pound per square inch. Values are represented as mean ± SD

Significance ****p < 0.0001 compared to sham and contralateral groups (Two-way ANOVA)

### Effects of optogenetic stimulation on pain behaviors

Optogenetic modulation of the vlPAG alters the behavioral pain scores in the neuropathic pain model group. TN group receiving the optogenetic virus (TN/ChR2) exhibited significant changes in the air puff test, and improved pain sensitivity was observed during the laser on condition (laser off = 5.33 ± 0.471 psi vs laser on = 18.33 ± 1.25 psi, ordinary two-way ANOVA, F (3,72) = 63.44, *p* < 0.0001, Fig. 3e). We also noticed significant differences in the acetone drop test during the laser on condition in TN/ChR2 rats (laser off = 27 ± 1.25 vs. laser on = 10 ± 0.47, two-way AVOVA, F (3,72) =119.2, *p* < 0.0001, Fig. 3f). The number of responses per minute declined during the optogenetic stimulation condition in TN/ChR2 rats, which indicated reduced pain sensitivity whereas no changes were observed in sham and TN/EYFP animals (Table 2).

**Table 2:** Effect of vlPAG optogenetic activation in mechanical and thermal pain sensitivity on trigeminal facial area

	*Laser off*	*Laser on*
*Air puff threshold (psi)*		
*TN/ChR2*	5.33 ± 0.47	18.33 ± 1.25****
*TN/EYFP*	6.67 ± 2.05	7.11 ± 1.63****
*Sham/ChR2*	21.11 ± 1.25	20.13 ± 2.32
*Sham/EYFP*	20.21 ± 3.10	21.23 ± 1.10
*Cold hyperalgesia (No. of responses)*		
*TN/ChR2*	27 ± 1.25	10 ± 0.47****
*TN/EYFP*	28 ± 1.63	26 ± 2.05****
*Sham/ChR2*	10 ± 2.0	9 ± 1.50
*Sham/EYFP*	8.5 ± 2.5	9.5 ± 1.32

Note: *TN* trigeminal neuralgi, *ChR2* channelrhodopsin, *EYFP* enhanced yellow fluorescent protein, *psi* pound per square inch, *TN/ChR2* TN group receiving optogenetic virus, *TN/EYFP* TN group receiving null virus, *sham/ChR2* sham group receiving optogenetic virus, *sham/EYFP* sham group receiving null virus; *n* = 10 per group; Values are represented as mean ± SD

Significance ****p < 0.0001 compared to laser on and off conditions (Ordinary two-way ANOVA)

### Optogenetic stimulation of vlPAG inhibits thalamic activity

**Fig. 4:**
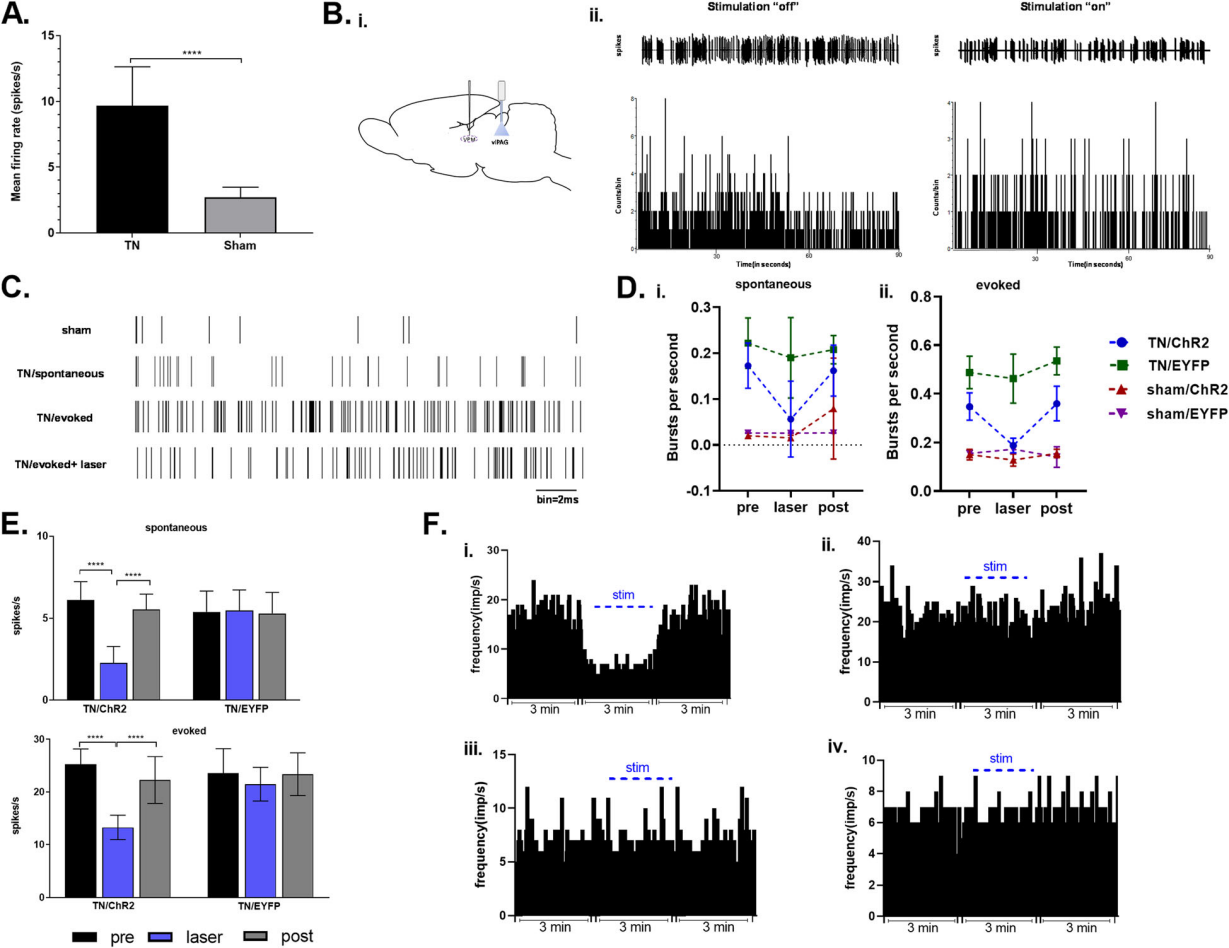
Electrophysiological findings from VPM thalamus under optogenetic stimulation of vlPAG. **a** Mean firing rates in VPM neurons in TN animals against sham animals (****, *p* < 0.0001, significant variation defined using unpaired t-tests). **b** i. Schematic diagram showing recording area and optic stimulation area. ii. Representative spike traces (above) and rate histogram (below) obtained from a TN/ChR2 rats in blue laser stimulation on and off conditions. **c** Representative raster traces recorded from VPM thalamus of TN and sham animals. **d** Spontaneous(i) and evoked (ii) in vivo *burst* recordings obtained from the VPM thalamus during different light conditions. **e** Spontaneous and evoked mean firing rate (expressed in spikes per second) from TN/ChR2 and TN/EYFP rats in different laser conditions. **f** Representative firing frequencies (expressed as impulses per second) observed in animal groups (i. TN/ChR2, ii TN/EYFP, iii. Sham/ChR2, iv. sham/EYFP) through various light frame. ****, *p* < 0.0001, significant difference determined via two-way analysis of variance (ANOVA)

During the optogenetic stimulation of vlPAG, electrophysiological monitoring of neuronal activities in the ventral posteromedial (VPM) area of the thalamus was performed to investigate the ascending pain inhibition pathway, as this site in the thalamus is reported to be involved in the trigeminal pain processing pathway. In the thalamus, spontaneous and evoked responses to mechanical stimuli in rats were recorded with subsequent stimulation of the excitatory neurons expressing ChR2 in vlPAG. The neuropathic (TN) rats showed increased mean firing rate (9.68 ± 2.94 spikes/s) in the thalamus compared to the sham rats with 2.7 ± 0.79 spikes per second (Fig. 4a). In response to laser on and off conditions in the vlPAG (Figure Bi), the figure Bii shows the representative raw data of spiking activity (above) and rate histogram (below) in TN rats. In the representative raster scan of sham and TN rats, the effect of optogenetic stimulation of vlPAG excitatory neurons on neurons in the thalamus can be seen (Fig. 4c). Spontaneous and evoked burst firing rates were higher in the TN rats in contrast to sham rats. TN /ChR2 rats showed decline in burst firing activity during the optogenetic stimulation in vlPAG but no fluctuations were seen in TN/EYFP animals (Fig. 4d). While thalamic neurons were hypersensitive to noxious stimuli in both TN/ChR2 and TN/EYFP rats, laser stimulation significantly reduced the firing responses in the TN/ChR2 group to mechanical stimuli (Fig. 4e). The thalamic responses in TN/EYFP and sham groups were unaffected by optogenetic stimulation (Fig. 4f). Thus, optogenetic modulation can lead to pain inhibition by selectively enhancing the inhibition circuitry of PAG-RVM-thalamus in neuropathic rats. Our electrophysiological results reveal the light modulated neuronal activities in VPM thalamus in response to vlPAG neuronal activation in ChR2 transfected neuropathic rats (Table 3). When the opsins expressed on vlPAG were activated, the resulting responses in VPM thalamic neurons were varied in neuropathic rats compared to sham models, showing reductions in firing rate. We did not find any significant change in firing in the histogram generated from sham controls.

**Table 3:** Spontaneous and evoked firing rates of VPM thalamic neurons in response to optic stimulation of vlPAG neurons

	Tonic firing (spikes/s)		Burst firing (bursts/s)	
	*Spontaneous*	*Evoked*	*Spontaneous*	*Evoked*
***TN/ChR2***				
pre	6.13 ± 1.11	25.21 ± 2.94	0.173 ± 0.049	0.348 ± 0.056
laser	2.26 ± 1.01***	13.26 ± 2.32**	0.056 ± 0.083**	0.188 ± 0.030**
post	5.52 ± 0.949	22.26 ± 4.45	0.162 ± 0.056	0.360 ± 0.071
***TN/EYFP***				
pre	5.67 ± 0.465	23.54 ± 4.65	0.22 ± 0.055	0.488 ± 0.067
laser	1.90 ± 0.316****	21.46 ± 3.20****	0.19 ± 0.088**	0.463 ± 0.100***
post	6.098 ± 0.296	23.35 ± 4.05	0.208 ± 0.031	0.535 ± 0.057

Note: *TN* trigeminal neuralgia, *ChR2* channelrhodopsin, *EYFP* enhanced yellow fluorescent protein, *pre* pre-stimulation, *post* post-stimulation, *TN/ChR2* TN group receiving optogenetic virus, *TN/EYFP* TN group receiving null virus, *spikes/s* spikes per second, *bursts/s* bursts per second; *n* = 10 per group; All values are represented as mean ± SD

Significance at **p < 0.01, ***p < 0.001, ****p < 0.0001, compared to pre and post conditions with laser condition (Two-way ANOVA)

### Effect of optogenetic stimulation in extracellular GABA and glutamate

**Fig. 5:**
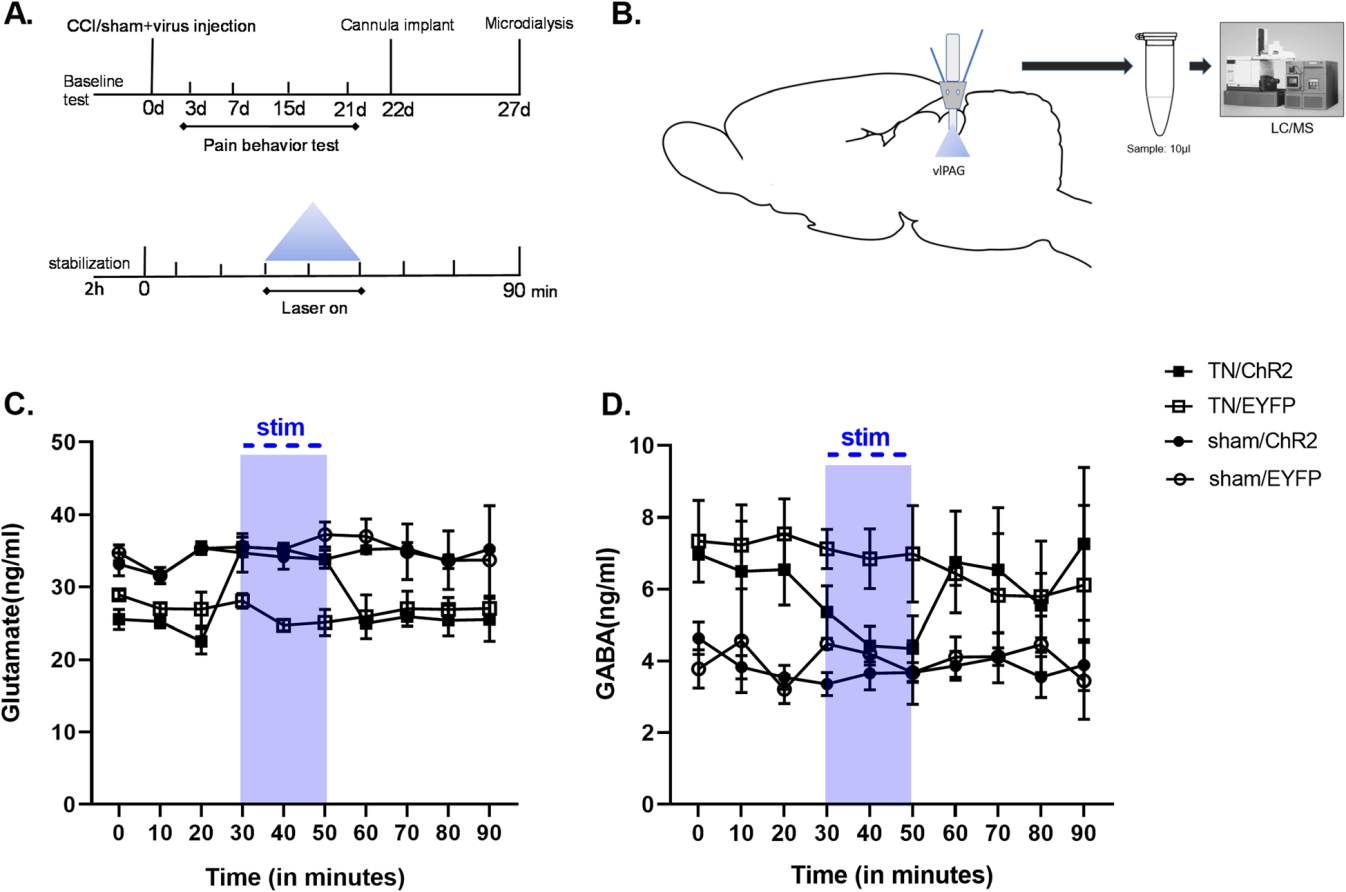
Microdialysis analysis at vlPAG. **a** Experimental timeline demonstrating virus injection and cannula implantation (above) followed by collection of samples by microdialysis (below). **b** Schematic diagram showing microdialysis probe implanted and stimulation area followed by liquid chromatography- mass spectrometry (LC/MS) analysis. Extracellular glutamate (**c**) and GABA (**d**) concentration at vlPAG in different animal groups at different laser conditions within the 90 min period. Blue shaded area indicates the optic stimulation period at vlPAG

Microdialysis probes were inserted into the vlPAG of TN and sham-operated rats to test the changes in neurotransmitter levels in response to optogenetic stimulation (Fig. 5a and b). TN rats displayed substantially lower levels of glutamate (25.61 ± 3.07 ng/ml) and increased GABA (7.17 ± 0.64 ng/ml) concentrations than rats treated with shams (glutamate:35.79 ± ng/ml and GABA:3.97 ± 0.54 ng/ml) in microdialysates collected from the vlPAG. In response to vlPAG optogenetic stimulation, TN/ChR2 rats displayed substantial decrease in GABA (5.08 ± 0.88 ng/ml) and increase in glutamate level (32.42 ± 1.26 ng/ml), but no variations were observed in TN/EYFP and sham controls (Fig. 5c and d). Studies have identified that pain facilitation could be the outcome of decreased excitatory neurotransmission in the vlPAG due to decreased glutamate release in vlPAG output neurons as the result of sustained increases in GABAergic neuronal activity [31]. In addition, animal studies have demonstrated that nerve injury-induced pain may be linked to descending pain modulation, signifying that dysregulation of descending inhibition represents a part of the transition from acute to chronic pain [11].

## Discussion

As an integral part of the neural pathway that mediates the sense of pain, ventrolateral periaqueductal gray (vlPAG) has been extensively studied [9, 31–33]. In steadiness with that, we found that activation of vlPAG neurons plays a vital role in pain behavioral responses and altered thalamic discharge in trigeminal neuralgia (TN) rat models with an induced chronic infraorbital nerve constriction (IoN-CCI) injury. Using optogenetic techniques, we observed that optogenetic activation of vlPAG glutamatergic neurons had pain mitigation outcomes. Microdialysate analysis and extracellular neural recording from the ventral posteromedial (VPM) thalamus under optogenetic stimulation on the vlPAG showed the functional association between these two regions in the trigeminal pain pathway. Together, these findings provide evidence establishing the important role of the vlPAG subneuronal population in the trigeminal neuropathic pain circuitry.

The midbrain PAG is one of the crucial brainstem area of ascending pain pathway that can significantly modulate incoming noxious inputs at the spinal trigeminal nucleus (SpV). The functioning of this brainstem region has attracted considerable attention for their potential role in the development and maintenance of chronic pain [34]. Via the trigeminal cranial nerve, noxious information from the face and mouth reaches the brainstem. In the spinal trigeminal tract, noxious afferent axons descend prior to synapsing in the SpV. Second-order nociceptive neurons in the rostral ventromedial medulla (RVM) cross the midline and ascend before synapsing into the thalamus in the trigeminothalamic tract. From the thalamus, for active pain perception, the message is relayed to different cortical regions. Second-order nociceptive neurons originating in SpV are also transmitted to PAG. The PAG, RVM, locus coeruleus project, either directly or indirectly, to modulate (inhibit or facilitate) nociceptive processing at the SpV as part of a pain-modulation network. A specific array of coping mechanisms has also been explored by stimulation of neuronal cell bodies in the vlPAG [35]. The PAG itself delivers minimal projections to the dorsal horn and SpV of the spinal cord and, rather, indirectly confers its impact by RVM projections [36]. Studies in rodents indicate that the PAG activates an inhibitory chain in the RVM and pontine noradrenergic nuclei through descending serotonergic and noradrenergic pathways, causing the sensation of nociceptive pain to abate. Another report has shown that morphine delivered to the PAG is able to trigger an ascending serotonergic pathway in the nucleus accumbens to release S-hydroxytryptamine, which in turn triggers the enkephalinergic system within the same nucleus, leading to an anti-nociceptive effect [37]. In addition to opioid- and serotonergic-produced analgesia, the PAG acts in both ascending and descending pain processing with glutamatergic and GABAergic neurons mediating opposing effects efferently through the descending pathway [38]. In general, our results are in accordance with a previous PAG study, which suggests that PAG electrical stimulation could impede trigeminal nociceptive feedback. Indeed, determining the precise neural mechanisms responsible for this outcome require further review.

Disruption of the descending analgesic network may be a contributing factor to the pathology of trigeminal neuropathic pain. Consistent with a previous report of the role of the PAG in TN pain modulation [39–41],we found that activation of the neuronal subpopulation in the vlPAG modulated TN pain behaviors in a rat model. Glutamatergic neurons in the vlPAG are the output neurons to the RVM; thus, optogenetic activation of these neurons might be effective in producing pain relieving effects. In TN patients, aberrant sensory input contributes to the transmission of irregular sensory feedback to the thalamus, causing a sensation of pain and behavioral changes [42] . In this study, compared to sham controls, TN rats exhibited significant reductions in pain behaviors in the air puff test and acetone drop test during stimulation of the vlPAG with a blue laser. Activation of glutamatergic neurons or inhibition of GABAergic neurons by chemogenetic approach results in significant inhibition of nociceptive sensation, according to recent research by Vijay K, despite the fact that the experiment was conducted in normal mice rather than a mouse pain model, contrast to our current study. Since our stimulation approaches and parameters were different from them, the discrepancy between the experiments may be linked to variations due to plastic changes in the neural circuitry. Moreover, we used different behavioral test in whisker pad to sensory input with the objective to determine the role of vlPAG in trigeminal pain circuitry which is also contrast with the previously reported studies.

Abnormal thalamic activity has been investigated in patients with neuropathic pain including TNP [43–45] as thalamus is a central node in the ascending pain pathway [46, 47]. It has been suggested that abnormal sensory symptoms in patients with chronic pain could be caused by impairment of the ascending and descending pain modulation circuits. Changes in the rhythmic burst firing of thalamic neurons can lead to the development of chronic pain following peripheral nerve injury. In each brainstem subnucleus, the somatotopic hierarchy of primary afferents is conserved by the trigemino-VPM pathway. From the brainstem to the thalamus, SpV relays somatosensory orofacial information. Via the caudo-rostral extent of the posterior triangular thalamic nucleus, spino-and trigemino-thalamic fibers run and then extend in the Po and VPM. Through the VPM and portion of the Po, the main trigeminal trajectory to the somatosensory cortex is reflective. Human functional magnetic resonance imaging (fMRI) studies of nociceptive neurotransmission has shown that the VPM nucleus is activated by thermal stimulation of trigeminal neurons and consistent thalamic activation was detected in human models of trigeminal nociceptive stimulation during spontaneous attacks [48, 49] Several previous studies have documented electrophysiological hyperresponsiveness of contralateral VPM in CCI-IoN rats, in line with our results. Our electrophysiological findings demonstrated the pathophysiologic shifts in thalamic activity as seen in diabetic neuropathic pain [50] and in sciatic nerve constriction injury cases [51], supporting the presence of a central neuropathic mechanism. We found significantly altered thalamic neuronal firing in the TN group, which is consistent with other reports suggesting heightened spontaneous activity, a wider receptive field size, a low activation threshold and, consequently, higher excitability in VPM neurons following trigeminal nerve injury [52]. However, the association of the VPM thalamus and PAG with TN has not been mentioned properly. Indeed, we observed VPM thalamic neuronal activity under optogenetic stimulation in the vlPAG and found reciprocally correlated activity in the trigeminal pain circuitry.

For homeostasis maintenance, the relative balance of GABAergic neurons and glutamatergic neurons is essential. When injury persists, a functional conflict between these neurons accompanies it. The CCI affects the inhibitory / excitatory balance of local inputs directly upstream of the PAG to medial prefrontal cortex (mPFC) neurons [53]. Previous studies noted the significance of vlPAG, which exerts both inhibitory and facilitative effects on pain perception [54, 55]. Via projections to the RVM, the vlPAG modulates spinal cord nociceptive transmission, establishing the PAG-RVM-spinal descending pain inhibitory network [56]. Glutamate and gamma-aminobutyric acid (GABA) are the major excitatory and inhibitory neurotransmitters in the PAG [57]. Altered balance of these neurotransmitters in brain regions such as the vlPAG occurs in chronic pain states and leads to augmented central pain processing and increased pain sensitivity. GABA interneurons are present throughout the rostrocaudal extension of the PAG and may account for approximately 36% of neuronal populations in the vlPAG and dlPAG columns. Notably, in trigeminal pain condition, reduced thalamic GABA levels were reported [58]. Earlier study demonstrated antinociceptive results by the microinjection of GABAA receptor antagonists or glutamate agonists into vlPAG. It is well documented that glutamatergic neurotransmission in vlPAG may indeed be crucial in neuropathic pain models to elicit a sustained decline in the descending inhibitory mechanism of pain, leading to chronic neuropathic pain. The decline in GABA levels was observed only in CCI animals in the current data, while sham rats did not display any variations in GABA levels during stimulation. It is well recognized that glutamatergic neurotransmission in the vlPAG can be critical in neuropathic pain models to cause a persistent reduction in the descending pain inhibitory system, which results in chronic neuropathic pain. The imbalance in GABA: glutamate ratio in TN/ChR2 rats was regulated with the optogenetic stimulation in vlPAG. Furthermore, our findings suggest that in sham models, glutaminergic neurotransmission in the PAG may not be essential as in TN models. These responses to vlPAG stimulation can also be influenced by the time course of the assessment or the methodological approach to the experimentation. The importance of using microdialysis is that this realistic approach assesses complex changes in the extracellular space that provide accurate GABA and glutamate measurements influenced by GABAergic afferents.

Markedly, there are some shortcomings of our study that deserve deliberation. For instance, animals did not receive any analgesics postsurgery to avert anticipatory analgesic influences on the progress of neuropathic pain. In addition, there might be anesthetic drugs might impact the thalamic discharge during neural recording; however, we provoked ipsilateral whisker pads with von Frey filaments to avoid such influences. In glutamatergic neurons, the excitatory neuron-specific promoter calcium-calmodulin dependent protein kinase II (CaMKIIα) is specifically active to drive reporter gene expression. Thus, often used as marker for glutamatergic neuronal expression however, CamKIIα predominately targets excitatory populations which can be further segmented. Hence, the precise target on these subpopulations could be more effectual. Future studies utilizing functional neuroimaging or an enhanced electrophysiological study along with optogenetics will facilitate the clarification of the comprehensive functional association between the vlPAG and VPM thalamus in trigeminal pain circuitry.

## Conclusion

Our study suggests a functional association between the vlPAG and medial thalamus in relation to trigeminal neuropathic pain. The results indicated that optogenetic stimulation of the excitatory neurons in vlPAG excites output neurons to RVM and to trigeminal dorsal horn resulting in disinhibition followed by activation of descending pain inhibitory pathway. Our findings open the possibility to target the neuronal population in the vlPAG which could be highly promising direction in management of trigeminal neuropathic pain.

## Data Availability

Data can be made available upon request.

## Abbreviations

TN: Trigeminal neuralgia
vlPAG: Ventrolateral periaqueductal gray
VPM: Ventral posteromedial
ANOVA: Analysis of variance
DBS: Deep brain stimulation
GABA: Gamma-Aminobutyric acid
IoN-CCI: Infraorbital nerve chronic constriction injury
AAV: Adeno-associated virus
hChR2: Human channelrhodopsin
CaMKII: Ca2+/calmodulin-dependent protein kinase II
EYFP: Enhanced yellow fluorescent protein
AP: Anterio-posterior
ML: Medio-lateral
DV: Dorso-ventral
LC-MS: Liquid chromatography–mass spectrometry
HPLC: High-performance liquid chromatography
ESI: Electrospray ionization
PFA: Paraformaldehyde
PBS: Phosphate buffer solution
OCT: Optimum cutting temperature
DAPI: 4′,6-diamidino-2-phenylindole
SD: Standard deviation
psi: Pound per square inch
RVM: Rostral ventromedial medulla
SpV: Spinal trigeminal nucleus
fMRI: Functional magnetic resonance imaging
mPFC: Medial prefrontal cortex
dlPAG: Dorsolateral periaqueductal gray

## References

[1] Bista P, Imlach WL (2019) Pathological Mechanisms and Therapeutic Targets for Trigeminal Neuropathic Pain. Medicines 6(3) 10.3390/medicines6030091 PMC6789505 31443547

[2] Niwant P, Motwani M, Naik S (2015). Atypical trigeminal neuralgia secondary to meningioma. Case Rep Dent.

[3] Uddin O, Studlack P, Akintola T, Raver C, Castro A, Masri R, Keller A (2018). Amplified parabrachial nucleus activity in a rat model of trigeminal neuropathic pain. Neurobiol Pain.

[4] Kitagawa J, Takeda M, Suzuki I, Kadoi J, Tsuboi Y, Honda K, Matsumoto S, Nakagawa H, Tanabe A, Iwata K (2006). Mechanisms involved in modulation of trigeminal primary afferent activity in rats with peripheral mononeuropathy. Eur J Neurosci.

[5] Meunier A, Latrémolière A, Mauborgne A, Bourgoin S, Kayser V, Cesselin F, Hamon M, Pohl M (2005). Attenuation of pain-related behavior in a rat model of trigeminal neuropathic pain by viral-driven enkephalin overproduction in trigeminal ganglion neurons. Mol Ther.

[6] Behbehani MM (1995). Functional characteristics of the midbrain periaqueductal gray. Prog Neurobiol.

[7] Watson JC, Sandroni P (2016) Central neuropathic pain syndromes. Mayo clinic proceedings, 91(3). Elsevier, p 372-385. 10.1016/j.mayocp.2016.01.017. 10.1016/j.mayocp.2016.01.017 26944242

[8] Samineni VK, Grajales-Reyes JG, Sundaram SS, Yoo JJ, Gereau RW (2019). Cell type-specific modulation of sensory and affective components of itch in the periaqueductal gray. Nat Commun.

[9] Samineni VK, Premkumar LS, Faingold CL (2017). Neuropathic pain induced enhancement of spontaneous and pain evoked neuronal activity in the periaqueductal gray that is attenuated by gabapentin. Pain.

[10] Knight Y, Goadsby P (2001). The periaqueductal grey matter modulates trigeminovascular input: a role in migraine?. Neuroscience.

[11] Zhang Y, Mao Z, Pan L, Ling Z, Liu X, Zhang J, Yu X (2018). Dysregulation of pain-and emotion-related networks in trigeminal neuralgia. Front Hum Neurosci.

[12] Ben-Haim S, Mirzadeh Z, Rosenberg WS (2018). Deep brain stimulation for intractable neuropathic facial pain. Neurosurg Focus.

[13] Iseppon F, Arcangeletti M (2020). Optogenetics and photopharmacology in pain research and therapeutics. STEMedicine.

[14] Deisseroth K (2015). Optogenetics: 10 years of microbial opsins in neuroscience. Nat Neurosci.

[15] Kalanithi PS, Henderson JM (2012) Optogenetic neuromodulation. International review of neurobiology, vol 107. Elsevier, p 185–205. 10.1016/B978-0-12-404706-8.00010-3. 10.1016/B978-0-12-404706-8.00010-3 23206683

[16] Knight YE (2002) Midbrain periaqueductal grey modulation of trigeminal nociception: Relationship to migraine. Doctoral dissertation, UCL (University College London). eprint:1010390.

[17] Deseure K, Hans GH (2015) Chronic constriction injury of the Rat's Infraorbital nerve (IoN-CCI) to study trigeminal neuropathic pain. J Vis Exp (103):e53167 10.3791/53167 PMC4692620 26437303

[18] Kernisant M, Gear RW, Jasmin L, Vit J-P, Ohara PT (2008). Chronic constriction injury of the infraorbital nerve in the rat using modified syringe needle. J Neurosci Methods.

[19] Correia PA, Matias S, Mainen ZF (2017) Stereotaxic adeno-associated virus injection and cannula implantation in the dorsal raphe nucleus of mice. Bio Protoc 7(18):e2549. 10.21769/BioProtoc.2549 10.21769/BioProtoc.2549 PMC5633075 29021994

[20] Paxinos G, Watson C (2006). The rat brain in stereotaxic coordinates: hard cover edition: Elsevier.

[21] Ahn DK, Lee SY, Han SR, Ju JS, Yang GY, Lee MK, Youn DH, Bae YC (2009). Intratrigeminal ganglionic injection of LPA causes neuropathic pain-like behavior and demyelination in rats. Pain.

[22] Moon HC, Heo WI, Kim YJ, Lee D, Won SY, Kim HR, Ha SM, Lee YJ, Park YS (2017). Optical inactivation of the anterior cingulate cortex modulate descending pain pathway in a rat model of trigeminal neuropathic pain created via chronic constriction injury of the infraorbital nerve. J Pain Res.

[23] Jeon HJ, Han SR, Park MK, Yang KY, Bae YC, Ahn DK (2012). A novel trigeminal neuropathic pain model: compression of the trigeminal nerve root produces prolonged nociception in rats. Prog Neuro-Psychopharmacol Biol Psychiatry.

[24] Ung K, Arenkiel BR (2012) Fiber-optic implantation for chronic optogenetic stimulation of brain tissue. J Vis Exp. (68):e50004. 10.3791/50004 10.3791/50004 PMC3490315 23128465

[25] Pawela C, DeYoe E, Pashaie R (2016) Intracranial injection of an optogenetics viral vector followed by optical cannula implantation for neural stimulation in rat brain cortex. Methods in molecular biology (Clifton, N.J.), 1408. Springer, p 227–241. 10.1007/978-1-4939-3512-3_15. 10.1007/978-1-4939-3512-3_15 26965126

[26] Sidor MM, Davidson TJ, Tye KM, Warden MR, Diesseroth K, McClung CA (2015). In vivo optogenetic stimulation of the rodent central nervous system. J Vis Exp.

[27] Zapata A, Chefer VI, Shippenberg TS (2009). Microdialysis in rodents. Curr Prot Neurosci.

[28] Chefer VI, Thompson AC, Zapata A, Shippenberg TS (2009). Overview of brain microdialysis. Curr Protoc Neurosci.

[29] Gage GJ, Kipke DR, Shain W (2012). Whole animal perfusion fixation for rodents. J Vis Exp.

[30] Vos BP, Strassman AM, Maciewicz RJ (1994). Behavioral evidence of trigeminal neuropathic pain following chronic constriction injury to the rat's infraorbital nerve. J Neurosci.

[31] Ho Y-C, Cheng J-K, Chiou L-C (2013). Hypofunction of glutamatergic neurotransmission in the periaqueductal gray contributes to nerve-injury-induced neuropathic pain. J Neurosci.

[32] Mills EP, Di Pietro F, Alshelh Z, Peck CC, Murray GM, Vickers ER, Henderson LA (2018). Brainstem pain-control circuitry connectivity in chronic neuropathic pain. J Neurosci.

[33] Knight YE, Bartsch T, Kaube H, Goadsby PJ (2002). P/Q-type calcium-channel blockade in the periaqueductal gray facilitates trigeminal nociception: a functional genetic link for migraine?. J Neurosci.

[34] Henderson LA (2018). Trigeminal neuropathic pain: evidence of central changes from human brain imaging investigations. Aust Endod J.

[35] Berton O, Covington HE, Ebner K, Tsankova NM, Carle TL, Ulery P, Bhonsle A, Barrot M, Krishnan V, Singewald GM (2007). Induction of ΔFosB in the periaqueductal gray by stress promotes active coping responses. Neuron.

[36] Heinricher MM (2016) Pain modulation and the transition from acute to chronic pain. Advances in experimental medicine and biology, 904, Springer, p 105-115. 10.1007/978-94-017-7537-3_8 10.1007/978-94-017-7537-3_8 26900066

[37] Morgan MM, Clayton CC, Boyer-Quick JS (2005). Differential susceptibility of the PAG and RVM to tolerance to the antinociceptive effect of morphine in the rat. Pain.

[38] Wu D, Wang S, Stein JF, Aziz TZ, Green AL (2014). Reciprocal interactions between the human thalamus and periaqueductal gray may be important for pain perception. Exp Brain Res.

[39] Moisset X, Villain N, Ducreux D, Serrie A, Cunin G, Valade D, Calvino B, Bouhassira D (2011). Functional brain imaging of trigeminal neuralgia. Eur J Pain.

[40] De Tommaso M, Vecchio E (2013). Primary headaches and trigeminal neuralgia: neuropathic pain yes or not? Evidences from neurophysiological procedures. Expert Rev Neurother.

[41] DeSouza DD, Moayedi M, Chen DQ, Davis KD, Hodaie M (2013). Sensorimotor and pain modulation brain abnormalities in trigeminal neuralgia: a paroxysmal, sensory-triggered neuropathic pain. PLoS One.

[42] Becerra L, Morris S, Bazes S, Gostic R, Sherman S, Gostic J, Pendse G, Moulton E, Scrivani S, Keith D (2006). Trigeminal neuropathic pain alters responses in CNS circuits to mechanical (brush) and thermal (cold and heat) stimuli. J Neurosci.

[43] Youssef AM, Gustin SM, Nash PG, Reeves JM, Petersen ET, Peck CC, Murray GM, Henderson LA (2014). Differential brain activity in subjects with painful trigeminal neuropathy and painful temporomandibular disorder. PAIN®.

[44] Kishima H, Saitoh Y, Oshino S, Hosomi K, Ali M, Maruo T, Hirata M, Goto T, Yanagisawa T, Sumitani M (2010). Modulation of neuronal activity after spinal cord stimulation for neuropathic pain; H215O PET study. Neuroimage.

[45] Elina KC, Moon HC, Islam J, Kim HK, Park YS (2021) The Effect of Optogenetic Inhibition of the Anterior Cingulate Cortex in Neuropathic Pain Following Sciatic Nerve Injury. J Mol Neurosci, 71(3):638–50. 10.1007/s12031-020-01685-7 10.1007/s12031-020-01685-7 32808249

[46] Monconduit L, Villanueva L (2005). The lateral ventromedial thalamic nucleus spreads nociceptive signals from the whole body surface to layer I of the frontal cortex. Eur J Neurosci.

[47] Apkarian AV, Hashmi JA, Baliki MN (2011). Pain and the brain: specificity and plasticity of the brain in clinical chronic pain. Pain.

[48] Bernstein C, Burstein R (2012). Sensitization of the trigeminovascular pathway: perspective and implications to migraine pathophysiology. J Clin Neurol.

[49] Henderson LA, Macefield VG (2013). Functional imaging of the human brainstem during somatosensory input and autonomic output. Front Hum Neurosci.

[50] Fischer TZ, Tan AM, Waxman SG (2009). Thalamic neuron hyperexcitability and enlarged receptive fields in the STZ model of diabetic pain. Brain Res.

[51] Zhao P, Waxman SG, Hains BC (2006). Sodium channel expression in the ventral posterolateral nucleus of the thalamus after peripheral nerve injury. Mol Pain.

[52] Doheny J (2020) Trigeminal neuropathic pain in rats: a role for thalamic hyperpolarization-activated cyclic nucleotide-gated channel activity. Doctoral dissertation, Boston University. https://hdl.handle.net/2144/41212

[53] Cheriyan J, Sheets PL (2018). Altered excitability and local connectivity of mPFC-PAG neurons in a mouse model of neuropathic pain. J Neurosci.

[54] Heinricher M, Tavares I, Leith J, Lumb B (2009). Descending control of nociception: specificity, recruitment and plasticity. Brain Res Rev.

[55] Hemington KS, Coulombe M-A (2015). The periaqueductal gray and descending pain modulation: why should we study them and what role do they play in chronic pain?. J Neurophysiol.

[56] Fields H (2004). State-dependent opioid control of pain. Nat Rev Neurosci.

[57] Lü N, Han M, Yang Z-L, Wang Y-Q, Wu G-C, Zhang Y-Q (2010). Nociceptin/Orphanin FQ in PAG modulates the release of amino acids, serotonin and norepinephrine in the rostral ventromedial medulla and spinal cord in rats. PAIN®.

[58] Bathel A, Schweizer L, Stude P, Glaubitz B, Wulms N, Delice S, Schmidt-Wilcke T (2018). Increased thalamic glutamate/glutamine levels in migraineurs. J Head Pain.

